# Supplementary material for: Fecal Supernatants from Patients with Crohn’s Disease Induce Inflammatory Alterations in M2 Macrophages and Fibroblasts
Source: Cells. 2023 Dec 27;13(1):60. doi: 10.3390/cells13010060 (PMC10777926; doi:10.3390/cells13010060)
Supplement: Supplementary file 1 [file cells-13-00060-s001.zip › cells-2760969-supplementary.pdf]

Supplementary material

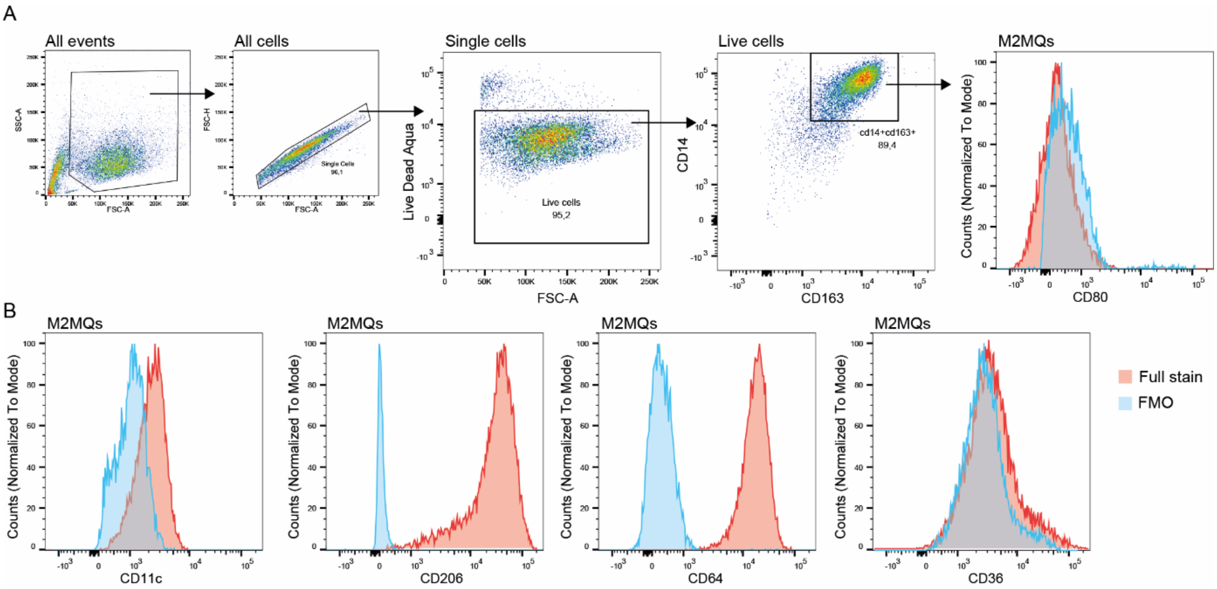

Supplementary Figure S1. Representative flow cytometry gating strategy for M2 macrophages (M2MQs). (A) From all events, all cells were selected, single cells were selected using FSC-A and FSC-H, live cells were defined as "LiveDeadAqua" stain negative and M2MQs were selected as being CD14^{high}CD163^{high}. Negative CD80 expression on M2MQs are shown to the far right. (B) Histograms showing expression of CD11c, CD26, CD64 and CD36. Pink colour shows stained cells and blue colour shows negative control (fluorescence minus one; FMO).

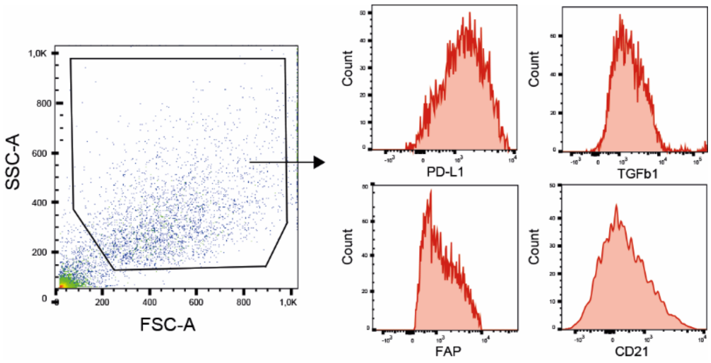

Supplementary Figure S2. Representative flow cytometry gating strategy for fibroblasts. Cells were selected (left) and median fluorescence intensity (MFI) was defined for the markers PD-L1, TGFb1, FAP and CD21.
